# Disconnected Pancreatic Duct Syndrome: A Rare Complication of Pancreatitis

**DOI:** 10.7759/cureus.61894

**Published:** 2024-06-07

**Authors:** Frank L Ventura, William C Lippert

**Affiliations:** 1 Internal Medicine, Wake Forest School of Medicine, Winston-Salem, USA

**Keywords:** general internal medicine, endoscopy ercp, disconnected pancreatic duct syndrome, pancreas disease, adult gastroenterology

## Abstract

Disconnected pancreatic duct syndrome (DPDS) is a rare complication of a common disease. Typically, DPDS occurs in acute necrotizing pancreatitis (ANP), chronic pancreatitis, abdominal surgery, or trauma. We present a case of DPDS from acute non-necrotizing pancreatitis (ANNP).

A 41-year-old male with a history of alcohol use and prior AP presented with progressive, severe left-sided abdominal pain that was worse with movement. Labs revealed a lipase of 95 U/L (normal range 11-82 U/L). Computed tomography (CT) of the abdomen/pelvis (A/P) with IV contrast demonstrated a large left-sided pleural effusion, non-necrotic pancreatic pseudocysts, and a large subdiaphragmatic fluid collection. Thoracentesis of the pleural effusion revealed an amylase of 601 U/L confirming pancreatic etiology. A subsequent magnetic resonance cholangiopancreatography (MRCP) confirmed complex peripancreatic ascites, rapid subdiaphragmatic fluid accumulation, and a fistula from the pancreatic tail to retroperitoneum concerning for a rapidly dissecting pancreatic pseudocyst. He ultimately underwent endoscopic retrograde cholangiopancreatography (ERCP) with stent placement in the main pancreatic duct. His left-sided abdominal pain rapidly improved, and the patient was discharged. CT A/P one week after discharge showed a reduced size of subdiaphragmatic fluid collection.

DPDS is usually seen in patients with a history of ANP. Our case demonstrates that it can also occur in ANNP, which has not previously been described in the literature. Therefore, a high index of clinical suspicion must be maintained for DPDS even in ANNP given its potential for severe complications.

## Introduction

Disconnected pancreatic duct syndrome (DPDS) is defined as a portion of the pancreatic duct that is disconnected from the proximal portion of the pancreas [1]. It most commonly occurs in cases of acute necrotizing pancreatitis (ANP) with a prevalence between 10% and 75% [1,2]. It has been previously reported as sequelae of chronic pancreatitis, trauma, post-pancreatic surgery, or malignancy, though its prevalence is lower in these cases compared to ANP [2]. Even in these more established scenarios, DPDS is frequently overlooked [1,2]. There is minimal literature describing disconnected pancreatic ducts in cases without necrosis. This case brings attention to the fact that DPDS can occur after acute non-necrotizing pancreatitis (ANNP), a review of its presenting symptoms, and its workup and management.

## Case presentation

**Figure 1:**
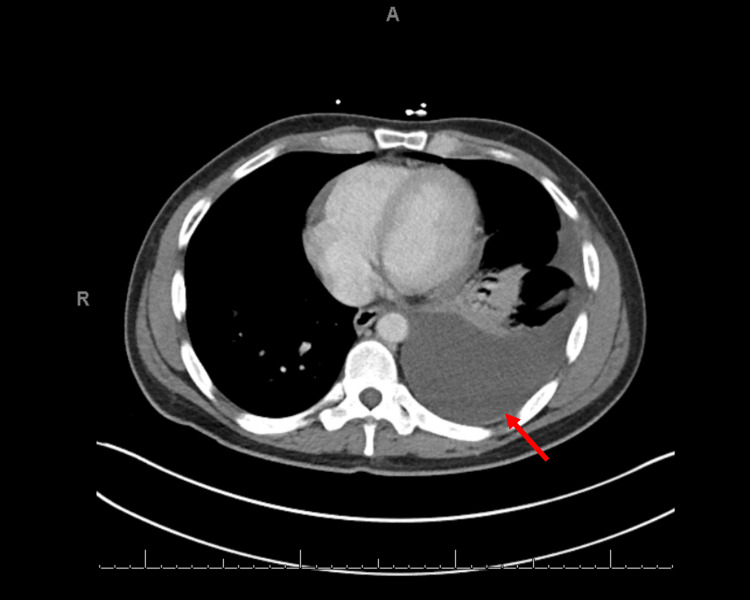
Computed tomography of the chest demonstrating a large left pleural effusion (red arrow) noted on admission.

**Figure 2:**
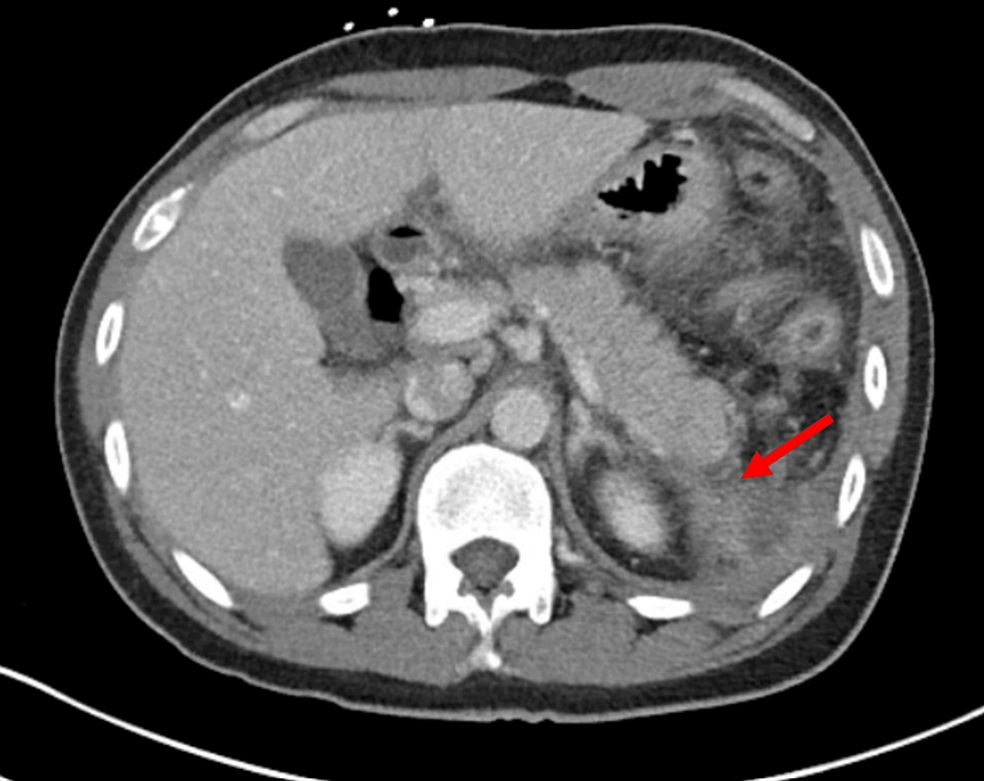
Computed tomography of the abdomen showing an accumulation of a subdiaphragmatic fluid collection (red arrow) on admission.

A 41-year-old male with a history of alcohol use, gastroesophageal reflux disease, and ANNP (two months prior) presented with progressive, severe left-sided abdominal pain that was worse with movement. He had no associated nausea, vomiting, diarrhea, constipation, shortness of breath, back pain, or fevers. Initial vitals demonstrated a heart rate of 104 with normal temperature and blood pressure. Labs were significant for a slightly elevated lipase at 95 U/L (normal range 13-78 U/L) and otherwise unremarkable blood work. Chest X-ray showed a left-sided pleural effusion. Computed tomography (CT) of the abdomen/pelvis (A/P) re-demonstrated a large left-sided pleural effusion (Figure 1) but also showed progressive pancreatic pseudocysts without necrotic features and a 4.3×2.5×2.4 cm subdiaphragmatic fluid collection (Figure 2). A thoracentesis was performed revealing an elevated amylase of 601 U/L, an exudative effusion per Light's criteria (lactate dehydrogenase ratio >0.6 and protein ratio >0.5), and pleural fluid cultures positive for *Bacillus pumilus* [3]. This information was suggestive of a pancreatic etiology of the pleural effusion. To treat the *Bacillus* species, he was initiated on ciprofloxacin for a planned 14-day course.

**Figure 3:**
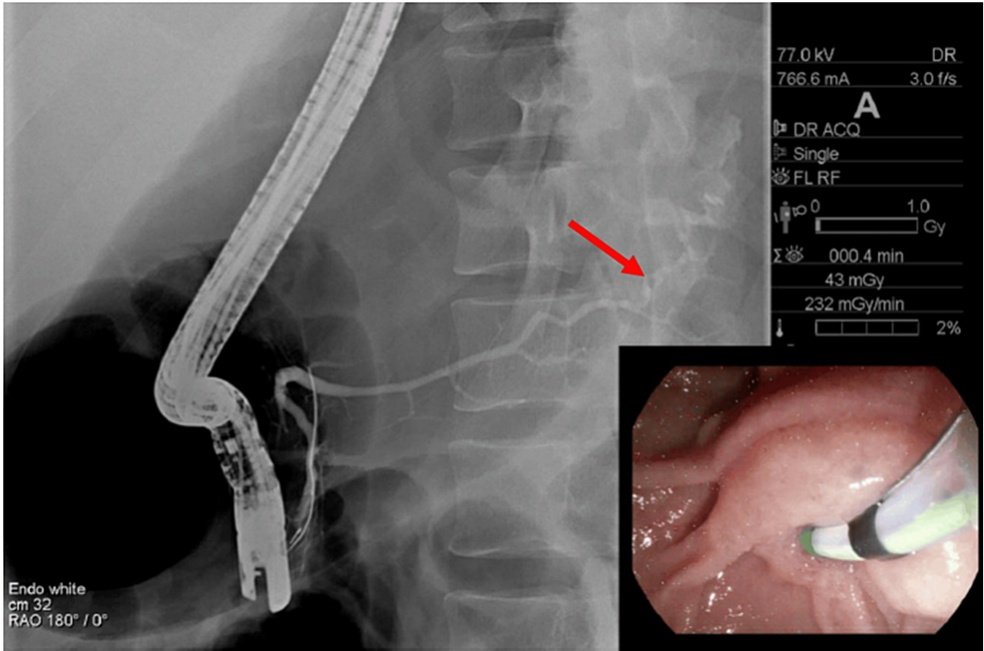
Endoscopic retrograde cholangiopancreatography demonstrating extravasation of contrast from the pancreatic tail (red arrow).

A subsequent magnetic resonance cholangiopancreatography (MRCP) confirmed complex peripancreatic fluid collections in the retroperitoneum, rapid accumulation of fluid in the left subdiaphragmatic region (14.2×3.6×7.2 cm), and direct communication from the pancreatic tail to the retroperitoneum concerning for a rapidly dissecting pancreatic pseudocyst and DPDS. He ultimately underwent a diagnostic and therapeutic endoscopic retrograde cholangiopancreatography (ERCP). Upon contrast injection, the ERCP noted extravasation from the pancreatic duct tail confirming DPDS (Figure 3). A pancreatic sphincterotomy/single pigtail stent placement was performed by gastroenterology. The subdiaphragmatic collection was deemed undrainable by interventional radiology. His symptoms rapidly improved, and the patient was discharged. CT A/P one week post-discharge showed a reduced size of subdiaphragmatic fluid collections (2.1×2.4×2.9 cm).

## Discussion

DPDS is a rare complication and can occur from ANP, chronic pancreatitis, abdominal surgery, and/or trauma [4-6]. Of those, DPDS is more frequently observed in ANP, and in a study by Neoptolemos et al., they observed that none of their 89 patients with ANNP developed DPDS, confirming the rarity of DPDS in ANNP [4-7]. Table 1 outlines a brief summary of the etiology of DPDS in previously prescribed papers. Literature search was performed in PubMed as "Disconnected pancreatic duct AND etiology". There were 17 full-text articles available. Articles that highlighted the causality of DPDS were included in our table. Our case is the first to describe DPDS from ANNP.

**Table 1:** Brief literature search on the etiology of DPDS DPDS: disconnected pancreatic duct syndrome; ANP: acute necrotizing pancreatitis; CP: chronic pancreatitis

Reference	Year of publication	Country	Study type	# of DPDS cases	Etiology
Satyam et al. [4]	2022	India	Case series	2	ANP, trauma
Chen et al. [6]	2019	China	Prospective cohort	31	ANP, trauma
Furuya et al. [8]	2021	Japan	Case report	1	Iatrogenic
Solanki et al. [9]	2011	India	Case report	1	Iatrogenic
Thiruvengadam et al. [10]	2022	United States	Retrospective cohort	27	ANP
Pearson et al. [11]	2012	United States	Retrospective cohort	7	ANP
Meng et al. [12]	2022	China	Case series	3	ANP
Chong et al. [13]	2021	United Kingdom	Meta-analysis	1355	ANP, CP
Maatman et al. [14]	2020	United States	Observational study	285	ANP

Patients can present with a variety of sequelae depending on the location of the fistula. The large spectrum of symptoms and timing has made DPDS particularly challenging to diagnose [2]. Sequelae to be mindful of include peripancreatic ascites, pleural effusions, and relapsing pancreatitis [5,15]. Patients may also be asymptomatic for an extended duration of months to years which also increases diagnostic difficulty. However, if left untreated, more severe complications may occur including intra-abdominal sepsis, peripancreatic hemorrhage, and enzymatic autodigestion leading to end-organ failure [6,8].

Workup of DPDS generally starts with imaging such as CT A/P with contrast or MRCP, though endoscopic ultrasound may also be used. Currently, literature does not favor one image over the other, and ultimately, ERCP remains the gold standard for diagnosis. Common radiologic studies of DPDS typically note at least 2 cm of pancreatic necrosis, the presence of viable pancreatic tissues upstream of the disconnection, and extravasation of contrast from the main pancreatic duct [16].

The gold standard for diagnosis is ERCP [5,8]. Unlike CT A/P or MRCP, ERCP is better for delineating the pancreatic duct anatomy during contrast opacification. It is capable of identifying a complete versus partial ductal obstruction, in which only a complete obstruction is considered DPDS [2,17]. Due to the improved diagnostic capability of ERCPs, recent studies have begun to include its contrast extravasation as part of the diagnostic criteria for DPDS [17].

Management of DPDS may include conservative monitoring, endoscopic drainage with ERCP, surgical drainage, and in severe cases a partial pancreatectomy. Choice of management is largely symptom-dependent. In asymptomatic cases with subjectively small fluid collections noted, monitoring may be the most appropriate option. However, the current data on conservative management remains guarded for DPDS resolution, and there is no strict definition of how fluid collections are considered "small" or "large" [1,10]. For those who are symptomatic, drainage is usually required either endoscopically or surgically [9]. Though endoscopic drainage is being performed more frequently to avoid surgery, a multitude of patients will later require surgical resection at some point [10,18].

There is little literature on the success rates of different management options. One article by Nealon et al. in 2008 compared the conservative and operative success rates in DPDS patients to those with a normal pancreatic duct [19]. Out of 130 patients with DPDS, 0% of them had spontaneous resolution. There were severely lower success rates with percutaneous drainage (0% versus 63.6%) and a significantly higher need for operative debridement of necrotic tissue (84.5% versus 39.3%). Particularly, the post-operative rate of maintaining/developing a fistula was also elevated (85% versus 27.3%) [19]. Given such guarded success rates, conservative management let alone operative management does not resolve most cases of DPDS.

Our case not only highlights an overlooked complication of pancreatitis but also highlights the first described case of DPDS in a patient with ANNP. Most literature proposes that clinicians maintain clinical suspicion in cases of necrotizing pancreatitis (NP) [1]. We hope this case works to lower the threshold of suspicion for DPDS, as our patient did with non-necrotizing disease.

## Conclusions

DPDS is a rare complication of a common disease. While it is easy to overlook the disease and simply attempt to treat underlying pancreatitis, DPDS can have severe complications if misdiagnosed. These complications, if left untreated, may even lead to end-organ failure. Patients who develop DPDS often present with a history of ANP; however, our case demonstrates that it can also occur in ANNP. This case not only brings light to an all-too-often overlooked diagnosis but emphasizes the importance of maintaining a high index of clinical suspicion even in atypical cases.
